# The Adhesion of *Lactobacillus salivarius* REN to a Human Intestinal Epithelial Cell Line Requires S-layer Proteins

**DOI:** 10.1038/srep44029

**Published:** 2017-03-10

**Authors:** Ran Wang, Lun Jiang, Ming Zhang, Liang Zhao, Yanling Hao, Huiyuan Guo, Yue Sang, Hao Zhang, Fazheng Ren

**Affiliations:** 1 Beijing Advanced Innovation Center for Food Nutrition and Human Health, China Agricultural University, Beijing 100083, P. R. China; 2 School of Food and Chemical Engineering, Beijing Technology and Business University, Beijing 100048, P. R. China; 3 Beijing Higher Institution Engineering Research Center of Animal Product, Beijing 100083, P. R. China

## Abstract

*Lactobacillus salivarius* REN, a novel probiotic isolated from Chinese centenarians, can adhere to intestinal epithelial cells and subsequently colonize the host. We show here that the surface-layer protein choline-binding protein A (CbpA) of *L. salivarius* REN was involved in adherence to the human colorectal adenocarcinoma cell line HT-29. Adhesion of a *cbpA* deletion mutant was significantly reduced compared with that of wild-type, suggesting that CbpA acts as an adhesin that mediates the interaction between the bacterium and its host. To identify the molecular mechanism of adhesion, we determined the crystal structure of a truncated form of CbpA that is likely involved in binding to its cell-surface receptor. The crystal structure identified CbpA as a peptidase of the M23 family whose members harbor a zinc-dependent catalytic site. Therefore, we propose that CbpA acts as a multifunctional surface protein that cleaves the host extracellular matrix and participates in adherence. Moreover, we identified enolase as the CbpA receptor on the surface of HT-29 cells. The present study reveals a new class of surface-layer proteins as well as the molecular mechanism that may contribute to the ability of *L. salivarius* REN to colonize the human gut.

Adhesion to intestinal epithelial cells is considered the first step in the persistent colonization of the host by *Lactobacillus* strains, which benefits the health of the host^1^. High-affinity adhesion promotes the residence of *Lactobacillus* in the host’s gut, excludes pathogens and protects epithelial cells^2^,^3^. Bacterial surface (S-) layers are crystalline arrays of self-assembling subunits called surface-layer (S-layer) proteins, and the S-layers form the outermost component of the cell walls of many species of eubacteria and archaea^4^. The S-layer is therefore one of the first bacterial components to interact with the gastrointestinal surface of the human host. Moreover, diverse bacterial cell surface-associated factors mediate specific adhesion and may act as adhesins such as carbohydrates, lipoteichoic acids as well as proteinaceous factors LPXTG-like protein^5^,^6^,^7^,^8^,^9^,^10^.

Since the adhesins have been identified, the mechanisms of adhesion are currently under intensive investigation. Numerous targeting strategies to identify the mechanisms of bacterial colonization of their host have been employed because of the multiple cell surface-associated factors expressed by lactobacilli. For example, Reunanen and Ossowski *et al*. found that the adhesion of *Lactobacillus rhamnosus* GG during intestinal colonization requires a pilus-mediated mucosal adhesin and a mucus-specific surface adhesin^11^,^12^. Certain *Lactobacillus* strains bind molecules such as mannose^13^, rat colonic mucins^14^, or glycolipids^15^. Therefore, adhesion likely does not require a unique and ubiquitous mechanism.

S-layer proteins form monomolecular crystalline arrays with molecular masses ranging from 40–200 kDa^1^, encompass the entire cell and form a regularly ordered array with oblique (p1, p2), square (p4) or hexagonal (p3, p6) symmetry^1^. The adhesive properties of the S-layer proteins of *Lactobacilli* have been widely suggested^1^. Numerous studies show that the loss of the S-layer proteins from the bacterial surface caused by chemical treatment decreases adhesion to different target cells^16^,^17^,^18^,^19^, indicating that the S-layer proteins may be one of the most important factors that mediates bacterial adherence to host cells. While, only in a few instances, the mechanism of *Lactobacillus* S-layer proteins in adherence has been definitely shown.

For example, recombinant CbsA of *L. crispatus* JCM 5810^20^ and SlpB of *L. crispatus* K313^21^ bind collagen types I and IV. SlpA of *L. acidophilus* NCFM binds to the dendritic cell-specific ICAM-3-grabbing non-integrin receptor expressed by immature human dendritic cells^22^. Further, the S-layer proteins mediate the binding of bacterial cells to receptors such as fibronectin^23^ and laminin^24^ as well as to human epithelial cell lines^25^,^26^.

The primary structures of S-layer proteins from different species include two functionally independent regions that mediate the adherence to targets and anchor the S-layer subunit to the bacterial cell envelope^21^. Generally, *Lactobacillus* S-layer proteins are devoid of a surface-layer homology domain that anchors the S-layer to the cell wall peptidoglycan^21^. Instead, sequences with similarity to tyrosine/phenylalanine-containing carbohydrate-binding motifs or teichoic acid-binding motifs are present in the cell-wall binding domains of *Lactobacilli* S-layer proteins^1^,^21^. On the other hand, S-layer proteins are anchored to the cell wall through different binding mechanisms. The S-layer protein CbsA of *L. crispatus* JCM 5810 binds to lipoteichoic acids through electrostatic interactions^27^; however, SlpA of *L. brevis* ATCC 8287 binds to neutral polysaccharides through hydrogen bonding^28^. These regions most likely are exposed on the surface of the S-layer proteins, which vary and share little sequence similarity^1^. Because of difficulties in obtaining high-quality crystals for X-ray crystallography, detailed structural information on their structures is scarce.

*L. salivarius* REN is a novel strain isolated from the fecal samples of a healthy centenarian living in a “longevity village” in the Bama District (Guangxi, China), which is home to one of the largest groups of centenarians in the world. Sun *et al*. reported the complete genome sequence of *L. salivarius* REN and found several specific genes related to its functions, such as alpha-glycerophosphate oxidase gene absence in *L. salivarius* ATCC 11741, contributing to degrade 4-hydroxyaminoquinoline 1-oxide, which could damage DNA^29^. *L. salivarius* REN decreases 4-nitroquioline 1-oxide-induced genotoxicity *in vitro*^30^, and prevents oral^31^ and colorectal carcinogenesis^32^. Further, *L. salivarius* REN binds with high affinity to intestinal mucus and epithelial cells and survives and proliferates in the rat colon^33^. These studies highlight the potential role of *L. salivarius* REN in probiotic activities, although the detailed mechanism of adhesion of *L. salivarius* REN to intestinal cells is unknown.

In the present study, we identified and isolated choline-binding protein A (CbpA) as an S-layer protein of *L. salivarius* REN that we show is important for adhesion. Moreover, we determined the crystal structure of the C-terminal region of CbpA, conducted *in vitro* analyses of the adhesive role of *L. salivarius* S-layer proteins and identified the CbpA receptor.

## Results

### The important role of S-layer proteins in adhesion of *L. salivarius* REN to the human colorectal cancer cell line HT-29

**Figure 1:**
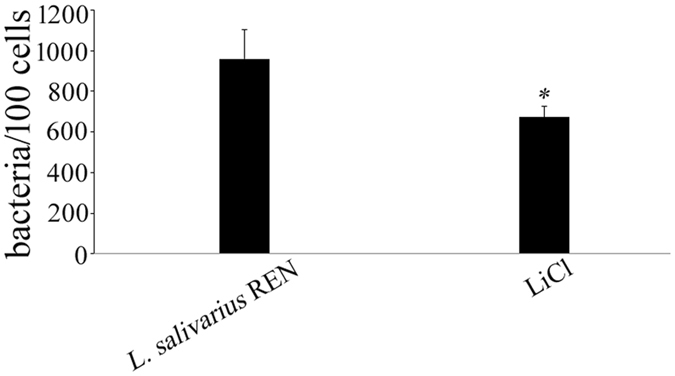
Adhesion of *L. salivarius* REN to HT-29 cells. The bars represent the mean values of three independent experiments, and the error bars indicate the standard deviations. Viable count of *L. salivarius* REN and LiCl-treated strains for adhesion to HT-29 cells were respectively 960 ± 90 and 673 ± 52 bacteria/100 cells. There was significant difference (*P < 0.05) in the adhesion of LiCl-treated *L. salivarius* REN compared with that of the untreated strains.

To assess the potential contribution of S-layer proteins to adherence, *L. salivarius* REN was treated with LiCl to remove S-layer proteins. The viable count of LiCl-treated bacteria was 3.8 × 10^9^ CFU/mL, indicating that good survival (85% livability) of the LiCl-treated cells was observed, and as previously reports^34^, treatment of *L. helveticus* with 5 M-LiCl led to a limited loss of viability (80%, i.e. <1 log unit). The adhesion value of this strain to HT-29 cells was 960 bacterial cells/100 HT-29 cells. However, after treatment with LiCl, a significant reduction (45%) in adherence was observed (Fig. 1). These results suggest that S-layer proteins play an important role in adherence to host cells.

### Isolation and identification of S-layer proteins of *L. salivarius* REN

**Figure 2:**
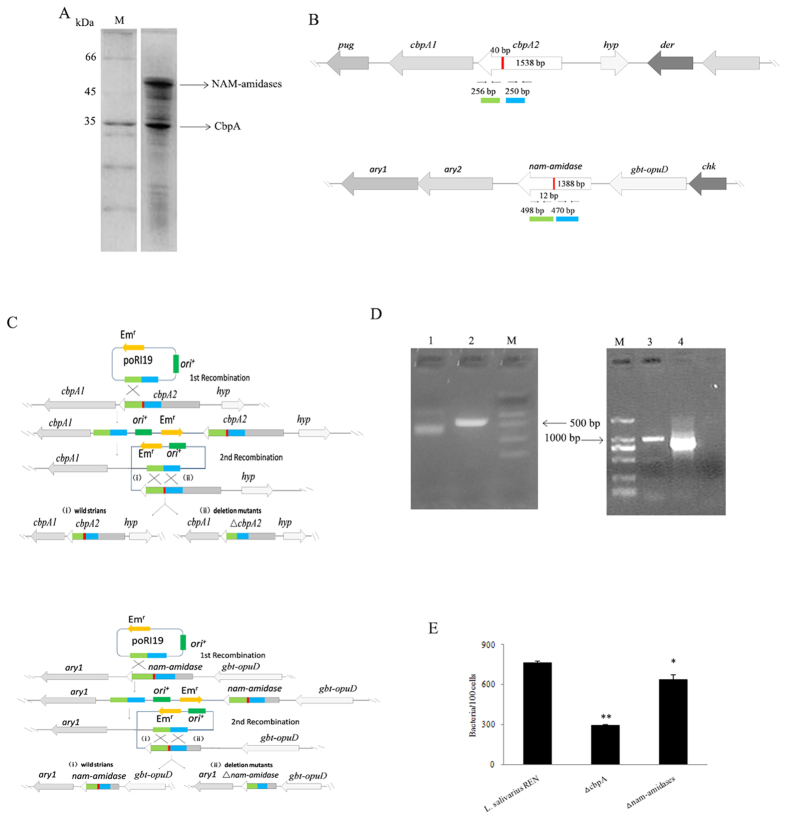
Identification of S-layer proteins of *L. salivarius* REN. (**A**) SDS-PAGE of cell surface proteins extracted from *L. salivarius* REN. (**B**) Gene organization of the region surrounding the *cbpA* and *nam*-*amidase* locus. Arrows indicate the locations of primers used for PCR analysis. The red shaded regions represented the deletion target. The green and blue shaded regions represented the upstream and downstream fragments in target genes. The chromosomal map is not drawn to scale. (**C**) Schematic overview of the construction of Δ *cbpA* and Δ *nam*-*amidase* mutants. (**D**) PCR identification of double crossover recombinant. Lane M: DNA marker DL 2000, lane 1: PCR amplification of *cbpA* in Δ *cbpA* mutants, lane 2: PCR amplification of *cbpA* in wild type strain, lane 3: PCR amplification of *nam*-*amidase* in wild type strain, lane 4: PCR amplification of *nam*-*amidase* in Δ *nam*-*amidase* mutants. (**E**) Adhesion of *L. salivarius* REN, Δ *cbpA* and Δ *nam*-*amidase* mutants to the HT-29 cells. Data were analyzed by the paired Student’s *t* test (*P < 0.05; **P < 0.001). Bars represented mean values of three independent experiments.

The S-layer proteins of *L. salivarius* REN were extracted with LiCl and separated using SDS-PAGE. Two dominant bands with approximate molecular masses of 35 kDa and 50 kDa were observed (Fig. 2A) and were provisionally designated SlpA and SlpB, respectively. MALDI-TOF-MS sequence analysis of the SlpA and SlpB bands revealed that SlpA was nearly identical (98%) to peptidase, specifically the M23 family of *L. salivarius* (Figure S2A). It was labeled as a domain of choline-binding protein A (CbpA, GenBank: AKI05012.1) in *L. salivarius* REN genome, which contains cell wall-anchoring choline binding domains (CWBD) in its N-terminal and C-terminal regions. SlpB was identical (99%) to the N-acetylmuramoyl-L-alanine amidase (NAM-amidase, GenBank: AKI04983.1) (Figure S2B). It was composed of a glucan-binding domain (YG repeat) at the N-terminal region and a peptidoglycan recognition protein (PGRP) domain at the C-terminal region.

To determine the roles of the S-layer proteins in adhesion, we constructed deletion mutants designated Δ*cbpA* and Δ*nam*-*amidase* and tested their ability to adhere to HT-29 cells. The adhesion values of Δ*cbpA* and Δ*nam*-*amidase* mutants to HT-29 cells were reduced by 62% and 16%, respectively, compared with wild-type (WT) (Fig. 2B), indicating that CbpA represents the major surface adherence factor of *L. salivarius* REN. Therefore, we focused our attention on CbpA.

### Crystal structure of CbpA

Analysis of the primary amino acid sequence of CbpA revealed that it contains a CWBD for anchoring to the surface of bacterial. The CWBD is characterized by the repetition of the 20-residue consensus motif GWX_6_WYYX_4_GXMX_2_ (G, Gly; W, Trp; Y, Tyr; M, Met; X, other residues)^35^, which comprises two highly conserved antiparallel β-strands connected by a short internal loop region^36^. The CWBD of Cbps binds noncovalently to choline moieties of teichoic and lipoteichoic acids, which are major components of the bacterial cell wall^36^. The CWBD of the S-layer protein of *L. acidophilus* ATCC 4356 mediates binding to the cell was as well^37^,^38^. Therefore, the C-terminal domain of CbpA (*DGL_654*, residues 338–512) may be responsible for adherence of bacteria to their host cells. This reasoning led us to solve the crystal structure of a truncated form of CbpA lacking the CWBD to identify the mechanism of adhesion.

**Figure 3:**
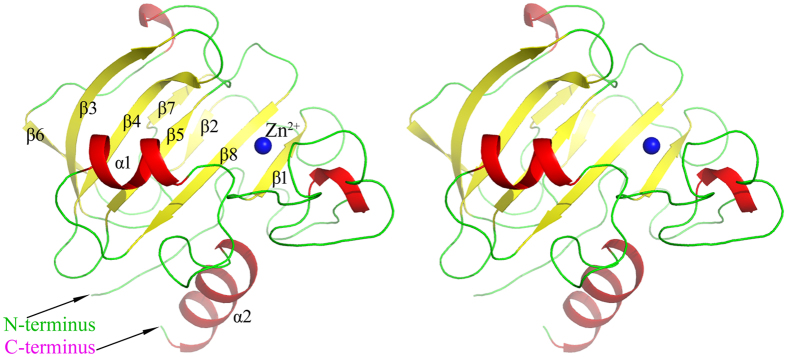
Stereoview of the CbpA structure. The β-sheets and α-helices of the entire structure are yellow and red, respectively. Zn^2+^ is shown as a blue sphere.

The structure of CbpA was solved at 1.85 Å resolution. One molecule was present in one asymmetric unit. The structure comprises residues 360–512, and residues 338–359 were not visible in the electron density map. The entire structure, which shares key features with those of M23-family peptidases^39^, includes a central six-stranded antiparallel β-sheet (β1, β3, β4, β5, β6 and β8) that forms a “sandwich” structure, two other β-sheets (β2 and β7) are packed against the bottom of the β-sheet and helices α1 and α2 in the C-terminus pack against the top (Fig. 3). The core antiparallel β-sheet anchors the catalytic residues, which bind a central Zn^2+^ (Fig. 3). The identification of Zn^2+^ was confirmed by crystallography (a decrease in the anomalous peak-height density at the metal ion sites passing from the absorption to the inflection-point wavelength) and was supported as well by similar temperature factors of the metal ion and the surrounding residues.

### The active site of CbpA

**Figure 4:**
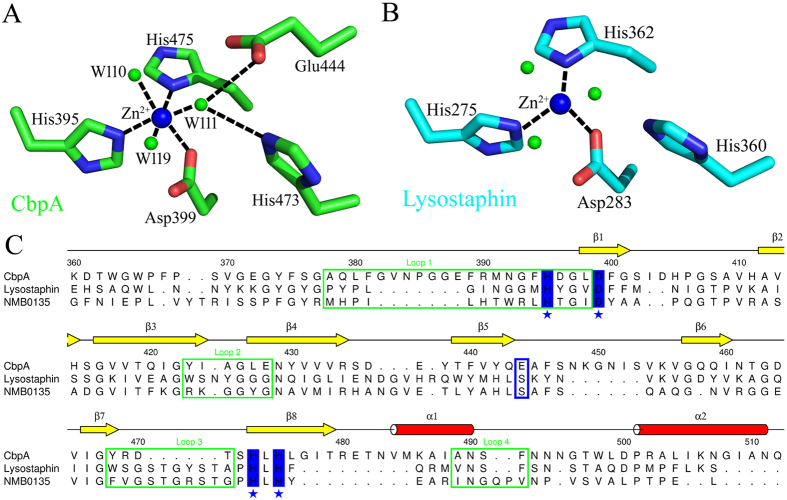
Catalytic sites of CbpA and lysostaphin. (**A**) Active sites in the crystals of CbpA (green) and (**B**) lysostaphin (cyan, PDB ID: 4QPB). The water molecules are shown as green spheres. (**C**) Sequence alignment of CbpA, lysostaphin and NMB0315 (Peptidase, M23 family of *Neisseria meningitidis* ATCC 13091, PDB ID: 3SLU). Secondary structure elements are represented according to the structure of CbpA. Residue numbers are labelled according to the sequence of CbpA. The four loops are highlighted in the green rectangle. The catalytic residues conserved among the three proteins are blue and denoted with asterisks. The Glu444 residue involved in the active site is colored blue in the rectangle.

Zn^2+^ is coordinated by three adjacent residues (His395, Asp399 of the characteristic HxxxD motif and His475 of the HxH motif) and three water molecules (W110, W111 and W119) in a nearly perfect octa-hedral arrangement. Glu444 and His473 interact with the metal ion through the W111 water molecule (Fig. 4A). The active site of CpbA (Fig. 4A) and Lysostaphin (Fig. 4B) are highly conserved, and the primary structures are also conserved among other M23-family peptidases (Fig. 4C).

### Structural comparisons of CbpA with other M23 metallopeptidases

**Figure 5:**
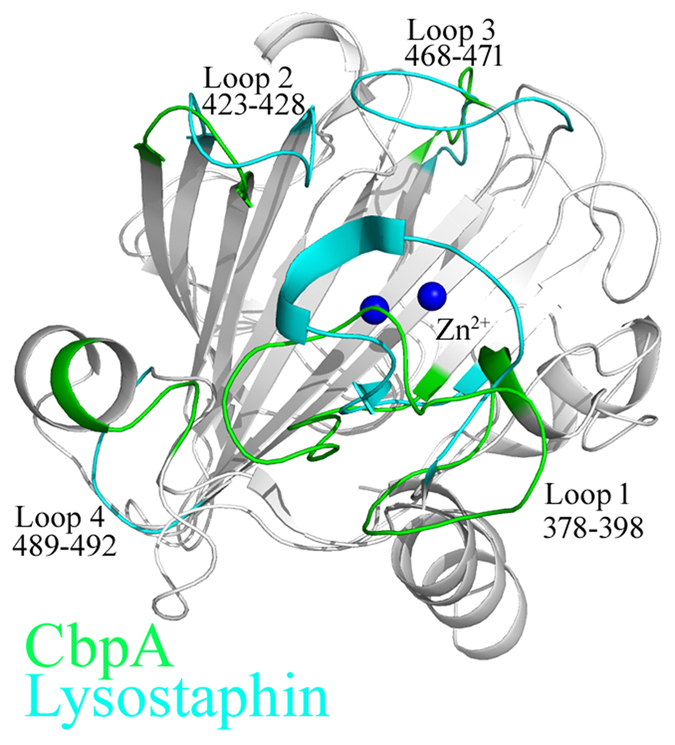
Structural alignment of CbpA and lysostaphin. The structures of CbpA (green) and lysostaphin (cyan) are analogous. The four different loops required for labeled with the amino acid residue numbers of CbpA.

Structural comparisons using the Dali Server^40^ revealed that the best match of CpbA was with lysostaphin from *Staphylococcus simulans* (PDB ID: 4QPB)^41^ with an overall root-mean-square deviation of 1.67 Å between 114 corresponding C-alpha atoms. Superimposition of CbpA onto lysostaphin (Fig. 5) revealed their similar overall topologies and connectivities. Each structure comprises a central six-stranded antiparallel β-sheet with the same spatial arrangement packed against β-sheets and α-helices to form a sandwich-like structure.

Although CbpA and lysostaphin share only 26% amino acid sequence similarity, their active sites are similar (Fig. 4B). The common active conformation of all M23 peptidase can be defined by the four loops (L1–L4) (Fig. 4C) that encompass the active site, which would be occupied by potential substrates (Fig. 5). The central six-stranded antiparallel β-sheet of CbpA forms the floor of the groove, and loops L1–L4 form the walls defined for lysostaphin^41^. CbpA contains a longer L-shaped Loop 1 above the Zn^2+^, thus forming a lid above the active site that regulates the entrance of the substrate towards the middle of the groove. The conformations of Loops 2 are similar in CbpA and lysostaphin, and the smaller Loop 3 in CbpA buries a smaller surface area that is accessible to the substrate. It differs most in the loop 4 of CbpA, forming an α-helix rather than a coil that is present in lysostaphin (Fig. 5). These different conformations of the loops in CbpA may indicate binding to more diverse substrates compared with lysostaphin.

### Identification of the HT-29 receptor for *L. salivarius* REN CbpA

**Figure 6:**
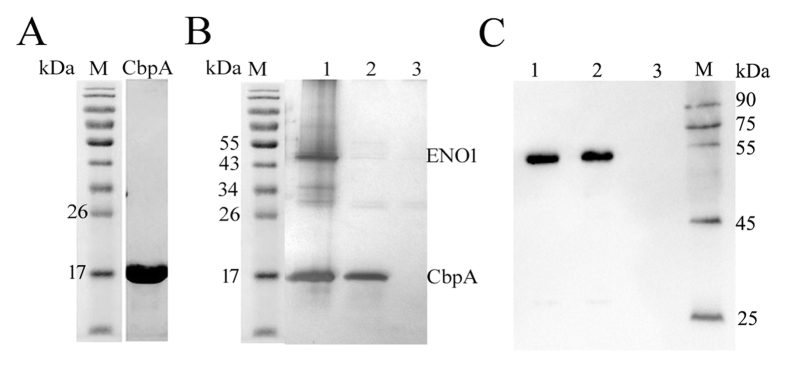
Enolase (ENO1), the putative CbpA receptor of HT-29 cells. (**A**) Recombinant CbpA was purified using Ni^2+^-agarose affinity chromatography. Lane M: marker. (**B**) The receptor of His-CbpA on HT-29 cells was identified using a His pull-down assay. Lane 1: bait protein (His-CbpA)-prey protein complex. Lane 2: bait protein (His-CbpA). Lane 3: control without bait protein. (**C**) Western blotting analysis with anti-ENO1 polyclonal antibodies. Extracts from HT-29 cells were co-immunoprecipitated by anti-CbpA antibody. Co-immunoprecipitated proteins were analyzed by Western blotting with anti-ENO1 polyclonal antibodies. Lane 1: the receptor of His-CbpA. Lane 2: the receptor of CbpA. Lane 3: the control agarose resin.

To identify the host-cell receptor that binds to *L. salivarius* REN, the M23 peptidase domain of CbpA (*DGL_654*) was expressed as a soluble His_6_-fusion protein in *E. coli* with high purity (Fig. 6A).

We performed pull-down assays of lysed HT-29 cells using a His-CbpA as bait. We detected an approximately 50 kDa band, which we designated “receptor of S-layer protein” (RlpA) (Fig. 6B). The sequence of RlpA, which we determined using MALDI-TOF-MS, is 98% identical to that of human enolase (ENO1, NCBI accession No.: NP_001419) (Figure S2C). To clarify the interaction between CbpA and ENO1, extracts of HT-29 cells were co-immunoprecipitated with His-CbpA (expressed in *E. coli*) or CbpA (extracted by LiCl treatment). When we probed western blots of the respective immunoprecipitates using an anti-ENO1 polyclonal antibody, we detected bands corresponding to the expected sizes of approximately 50 kDa (Fig. 6C).

## Discussion

The probiotic *L. salivarius* REN adheres to intestinal mucus and epithelial cells *in vivo*^33^. To study the molecular mechanism that sustains the ability of this bacterium to colonize the human gut and interact with the intestinal mucosa, S-layer proteins were removed by LiCl treatment, and the treated strain showed significant (*P* < 0.05) reductions in adhesion to HT-29 cells, suggesting that S-layer proteins play an important role in bacterial adherence to host cells.

We used SDS-PAGE and MALDI-TOF-MS to identify the S-layer proteins CbpA and NAM-amidase. Sequence analysis of CbpA revealed a CWBD motif that anchors CbpA to the bacterial envelope as well as an M23 peptidase sequence. The M23 peptidases of certain pathogens can act as adhesins. For example, *Treponema denticola* proteins with M23 peptidase domains that bind fibronectin^42^, and NlpD, which contains an M23 peptidase domain, is specifically required for intestinal colonization in an infant mouse model of cholera^43^. Further, deletion of the gene encoding the M23 peptidase family-member HdpA (HP0506) reduces the virulence of *Helicobacter pylori* by decreasing its ability to colonize its host^44^.

NAM-amidases of pathogens function as adhesins. These include AtlE of *Staphylococcus epidermidis*, which contains an amidase domain, mediates adherence to vitronectin and contributes to the binding of host cells during the stages of adherence^45^. The adhesin Aaa of *Staphylococcus aureus* contains a cysteine, histidine-dependent amidohydrolase/peptidase (CHAP) domain that binds in a concentration-dependent manner to fibrinogen, fibronectin and vitronectin and may promote the colonization of the host’s extracellular matrix and tissue^46^. However, adhesion mediated by M23 peptidases and NAM-amidases of probiotics is not assessed.

S-layer proteins can determine cell shape, and removal of the S-layer proteins may change the shape of a bacterial cell and thus alter its surface characteristics^47^. We show here that the abilities of Δ*cbpA* and Δ*nam*-*amidase* mutants to attach to HT-29 cells were significantly decreased compared with that of WT, suggesting that the S-layer proteins CbpA and NAM-amidase contribute to the ability of *L. salivarius* REN to bind to its host cells. Similarly, the abnormal shape of an *hp0506* mutant of *H. pylori* significantly inhibits its ability to colonize the gastric mucosa of mice^44^. Moreover, in the present study, the adhesive ability of the Δ*cbpA* mutant was significantly reduced compared with that of the Δ*NAM*-*amidase* mutant, suggesting a more important role in adhesion for CbpA.

We next solved the crystal structure of a C-terminal segment of CbpA that exhibited structural homology with M23 peptidase, indicating that CbpA is a protease. Evidence indicates that *Streptococcus pneumoniae* CbpG may represent a member of the S1 family of multifunctional surface-associated serine proteases and that its proteolytic activity may be required for adherence^48^. The structural similarities between the active sites of M23 metallopeptidases and CbpA indicate that the latter prefers glycine-containing peptides as substrates. Moreover, the differences in the conformation of its the loops compared with those of M23 metallopeptidases suggest that CbpA binds diverse substrates that are required for adherence. Molecular genetic experiments described above show that the adherence of CbpA required the presence of a proteolytic domain. Thus, CbpA may be a multifunctional surface protein that cleaves the host extracellular matrix and contributes to adherence.

Specific bacterial adhesion to intestinal cells depends on the receptor expressed on the host cell’s surface. The S-layer protein receptors expressed by intestinal epithelial cells are key factors in the interaction between gut microbiota and the host. Therefore, the identification of the receptors for S-layer protein is important for further research aimed to enhance the probiotic properties of lactobacilli. Here, the results of our *in vitro* binding and co-immunoprecipitation experiments show that enolase is the CbpA receptor expressed by HT-29 cells.

Enolase is a highly conserved glycolytic enzyme that represents a novel class of cell surface proteins. Enolase serves as a plasminogen receptor on the surface of a variety of hematopoietic, epithelial, and endothelial cells^49^. To determine the molecular mechanism of the interaction between CbpA and ENO1, we attempted to solve the structure of the complex formed by CbpA with ENO1. Despite repeated attempts to co-crystallize CbpA with ENO1, we failed to cocrystallized the proteins. Therefore, we conducted structural analyses of CbpA and ENO1.

Our experimental strategy was designed according to the findings of previous studies that demonstrate that the interaction between enolase and plasminogen increases the adherence of *S. pneumoniae* to epithelial and endothelial cells^50^. Therefore, we reasoned that CbpA mimics plasminogen in its interaction with ENO1, which mediates the adherence of *L. salivarius* REN to HT-29 cells. Further, the binding of enolase to plasminogen requires C-terminal lysine residues of enolase^51^. In the lysine binding sites of plasminogen, two adjacent aspartic acid residues coordinate the C-terminal lysine residues of enolase, and another arginine residue stabilizes the binding of the carboxylate moiety of the C-terminus of enolase^52^. Apart from these interactions, the methylene groups of the C-terminus of enolase are stabilized in the binding pocket through van der Waals contacts with the side-chain atoms of tryptophan residues^52^,^53^,^54^,^55^. In the active site of CbpA (Fig. 4A,C), the corresponding anionic center that coordinates the lysine residues of ENO1 may include Asp396 and Asp399, and the binding of the cationic to the carboxylate moiety of ENO1 may require Arg390. Further, the histidine residues in the active site may contribute to stabilizing the hydrophobic residues of ENO1. Moreover, the electrostatic potential map of the active site of CbpA reveals a continuous negatively charged surface, and that the surfaces of the C-terminal lysine residues of ENO1 are positively charged, indicating that they may interact with each other (Figure S1A,B). Multiple positively charged surfaces of ENO1 may participate in the interaction with CbpA as well.

In summary, we analyzed and identified two active S-layer proteins of *L. salivarius* REN (CbpA and NAM-amidase) and detected their different contributions to bacterial adhesion to HT-29 cells. We determined the crystal structure of the major surface adherence factor CbpA. CbpA may represent a new class of surface layer proteins, which contain an N-terminal domain required for cell wall-anchoring through choline binding and an M23 family peptidase C-terminal domain required for the interaction with the ENO1 receptor on the surface of host cells. Moreover, through structural analysis, we identified potential amino acid residues that mediate the binding of CbpA to ENO1. The interesting structural features of CbpA may be exploited for the development of selective activators or inhibitors to regulate the interaction between CbpA and ENO1 and enhance the beneficial effects of probiotics.

## Methods

### HT-29 cells cultures

HT-29 cells were obtained from Peking Union Medical College Hospital in Beijing, China and maintained in Hyclone DMEM/high glucose solution (Thermo Fisher Scientific, Waltham, MA, USA), with penicillin (50 U/mL), streptomycin (50 mg/mL) and 10% fetal bovine serum (Sigma-Aldrich, St. Louis, MO, USA) at 37 °C in an atmosphere of 5% CO_2_-95% air. For the adhesion assay, HT-29 cell monolayers were prepared on glass cover slips that were placed in 24-well tissue culture plates; the culture media was changed every other day. Cells were seeded at a concentration of 1 × 10^6^ cells/well. All experiments and cell maintenance were carried out at 37 °C in 5% CO_2_-95% atmospheric air.

### Bacterial adhesion to HT-29 cells

All bacterial strains and plasmids are listed in Table S1. Adhesion of *L. salivarius* REN to HT-29 cells was examined as previously described^56^. Briefly, bacterial cells were grown in de Man-Rogosa-Sharpe (MRS) broth (pH 6.5) under aerobic conditions at 37 °C to late exponential phase. Cultures were centrifuged at 4500× g for 10 min at room temperature and washed once, and then re-suspended in sterile phosphate-buffered saline (PBS) before cell density was adjusted to ~1 × 10^8^ CFU/mL based on OD_600_. HT-29 monolayers were washed twice with PBS. For each adhesion assay, 1 mL of *Lactobacillus* suspension was added to each well of the tissue culture plate at the multiplicity of infection (MOI) of 0–100, and incubated at 37 °C for 1 h in 5% CO_2_-95% atmospheric air. After incubation, the monolayers were washed five times with sterile PBS, fixed with methanol, Gram-stained, and examined using a light microscope. Each adhesion assay was duplicated using cells from three successive passages. For each monolayer on a glass cover slip, the number of adherent bacteria was counted in 20 random areas. *Lactobacillus* adhesion is expressed as the number of bacteria adhering to 100 HT-29 cells.

### Determination of the effect of LiCl

Viability was determined on MRS agar plates after incubation for 48 h at 37 °C as described by Lortal *et al*.^34^. The late exponential-phase cells (1.5 mg dry mass mL^−1^; 4.5 × 10^9^ CFU/mL) were incubated for 1 h at 37 °C with 5 M LiCl to extract the S-layer proteins. The treated bacteria were divided into two parts. One part was recovered by centrifugation, washed once in sterile distilled water and resuspended in the initial volume of MRS broth for the determination of viable bacteria by plate counting. Another was used for the adhesion assay. The bacterial pellet was washed once, and resuspended in sterile PBS before cell density was adjusted to ~1 × 10^8^ CFU/mL based on viable cell count and OD_600_.

### Isolation of bacterial cell surface proteins

S-layer proteins were extracted with 5 M LiCl as described by Lortal *et al*.^34^ using a slight modification. Briefly, the bacterial biomass from 500 mL of a culture was harvested by centrifugation (9000× g, 4 °C) and washed twice with sterile deionized water. One gram of wet cells was extracted with 5 mL of 5 M LiCl at 37 °C for 1 h. The cell suspension was centrifuged at 10,000× g for 10 min at 4 °C, and the supernatants were collected and dialyzed overnight in deionized water at 4 °C. Finally, the dialysate was freeze-dried and stored at −20 °C.

### Sequence analysis of S-layer proteins

The S-layer proteins of *L. salivarius* REN were separated using 12% (wt/vol) sodium dodecyl sulfate-polyacrylamide gel electrophoresis (SDS-PAGE). The bands on the gel were excised and sequenced using matrix-assisted laser desorption ionization time-of-flight mass spectrometry (MALDI-TOF-MS) (Beijing Bioms Sci-tech Co., Ltd., Beijing, China). The protein sequences were compared with *L. salivarius* REN sequence using blastp.

### Inactivation of genes encoding S-layer proteins

S-layer protein mutants were generated using a modified procedure described by van Pijkeren *et al*.^57^. Genomic DNA of *L. salivarius* REN was isolated using a genomic DNA purification kit (Tiangen Biotech Co., Ltd., Beijing, China). The genomic DNA of *L. salivarius* REN was used as a template for PCR amplification of the 5′-and 3′-end flanking regions of *DGL_654* and *DGL_683*, using the primer pairs AIE51F-AI52R, AI33F-AIB34R, BIE51F-BI52R and BI33F-BIB34R (Table S3). The amplicons were joined by splicing through overlap extension (SOE)-PCR using primer pairs AIE51F-AIB34R and BIE51F-BIB34R. The amplicons (500 bp and 910 bp) were digested using *Eco*RI and *Bam*HI, respectively, and ligated to pORI19. The integrity of each transformant was verified by digestion with *Eco*RI and *Bam*HI. The plasmids were designated pORS001 and pORS002, respectively. The plasmid integrants in *L. salivarius* REN were constructed with minor modifications described by Russell *et al*.^58^. Briefly, *L. salivarius* REN harbouring the helper plasmid pTRK669 was transformed with pORS001 or pORS002 by electroporation. Electroporation was carried out by using a MicroPulser (Bio-Rad Laboratories, Richmond, CA, USA). Pulse parameters were set as following: capacitance, 25 μF, voltage, 2.4 kV, and time, 4.5 ms. Then, the bacterial cells were immediately but gently suspended in 1 mL supplemented MRS medium and incubated at 37 °C for 2 h, then spreaded on MRS plates containing erythromycin (Em) and chloramphenicol (Cm), cultured for 48 h at 37 °C. Subsequently, two Em/Cm-resistant transformants carrying both plasmids were propagated 16 h once at 37 °C in MRS broth containing 5 μg/mL each of Em and Cm, and transferred three times (1% inoculum) in MRS broth with Em (ca. 30 generations) at 43 °C, with selection for pORS001 and pORS002 only, thus allowing integration into the chromosome upon the loss of pTRK669. The mutant genes encoding the S-layer proteins were confirmed using PCR with the primer pairs AIE51F-ORI19DF^57^ and BIE51F-ORI19DF. The adhesion of mutants to HT-29 cell lines was evaluated as described above.

### Protein Expression and Purification

The gene encoding residues 338–512 of CbpA (*DGL_654*) was cloned into a modified pET-28a vector, in which the thrombin recognition site is replaced by a tobacco etch virus (TEV) recognition site. The sequence of the insert was verified using DNA sequencing, and the plasmid was used to transform *E. coli* strain BL21 (DE3) for protein expression. Transformed cells were cultured in LB medium at 37 °C to an OD_600_ = 1.0, induced using 0.1 mM IPTG, and cultured further for 12 h at 18 °C. Cells were harvested by centrifugation at 3,000× g for 15 min, resuspended in lysis buffer (20 mM Hepes pH 7.0, 500 mM NaCl) and lysed using a sonicator. The lysate was centrifuged at 47,000× g for 20 min, the supernatant was filtered through a 0.45-μm filter membrane to remove cell debris and other impurities, and then applied to a Profinity IMAC Ni-Charged Resin column (Bio-Rad). To excise the 6× His tag, a small amount of 6× His-tagged TEV protease was added and incubated on ice overnight. The digestion products were applied to a Ni-Charged Resin column to remove TEV protease. The flow through was loaded onto a Superdex 200 HR10/300 GL column (GE Healthcare) equilibrated with 20 mM Hepes pH 7.0, 150 mM NaCl. The protein peak was identified using SDS-PAGE. The proteins were concentrated to 8 mg/mL for crystallization.

### Crystallization and Data Collection

Crystals of CbpA were grown at 18 °C using the sitting drop vapor diffusion method and equilibrated to a reservoir solution of 1.0 M (NH_4_)_2_HPO_4_, 0.1 M acetate, pH 7.5. After several days, the crystals were frozen in a cryoprotectant consisting of the reservoir solution containing with 20% ethylene glycol. The crystal data were collected at SSRF beamline BL17U and integrated and scaled using the HKL2000 suite^59^. Data collection statistics are summarized in Table S2.

### Structure determination and refinement

Using balbes (http://www.ccp4.ac.uk/ccp4online/) to perform searches, molecular replacement solutions of the CbpA were initially found. A crude partial model was built automatically using IPCAS (Iterative Protein Crystal structure Automatic Solution). The structure was completed using alternating cycles of manual model building (in coot ref. 60) and restrained refinement (in refmac5 ref. 61), and the final Rwork/Rfree was 17.2%/19.7%. All figures in this article displaying molecular structures created using PyMOL (The PyMOL molecular graphics system, version 1.3 r1, 2010).

### His pull-down assay of CbpA binding to molecules expressed by HT-29 cells

The His pull-down assay was performed using the Pierce His Protein Interaction Pull-Down Kit (Thermo-Fisher Scientific, Waltham, MA, USA). Briefly, His-CbpA served as bait and was incubated with HisPur Cobalt Resin at 4 °C for at least 30 min, the resin was washed with wash solution, and incubated with HT-29 cell lysates overnight at 4 °C. HT-29 cell lysates were prepared according to the user guide of the Pierce His Protein Interaction Pull-Down Kit. Briefly, HT-29 cells were released from the surface of the flask by Lifters, and harvested by centrifugation (500× g, 5 minutes), then washed once with 1 mL of Tris Buffered Saline (TBS) per 5 mL of original cell culture volume. HT-29 cell pellet was resuspended with 2.5 mL of ice-cold TBS per gram wet weight of cells, and added 5 mL of Pierce Lysis Buffer per gram wet weight of cells, and then incubated on ice for ~30 minutes. Supernatant was obtained by centrifuging at 12,000× g for 5 minutes. Finally, the resin was washed with elution buffer, and the elution buffer containing bait and prey proteins was analyzed using SDS-PAGE.

### Sequence analysis of potential CbpA receptors

The proteins in the pull-down elution buffer were separated using 12% (wt/vol) SDS-PAGE. The bands bound to His-CbpA were excised from the gel and sequenced using MALDI-TOF-MS. Protein sequences were compared with the sequences deposited in the Mascot database (http://www.matrixscience.com).

### Co-immunoprecipitation

Co-immunoprecipitation assays were performed using the Pierce Co-Immunoprecipitation Kit (Thermo-Fisher Scientific, Waltham, MA, USA). Briefly, an antibody against CbpA was coupled onto an amine-reactive resin at room temperature for 90 min, and then the CbpA–HT-29 cell lysate mixture was added to the immobilized antibody and incubated overnight at 4 °C. The resin was washed with elution buffer, and the proteins in the eluate were separated using SDS-PAGE. The proteins were electrophoretically transferred onto 0.2-μm Immobilon-P polyvinylidene difluoride (PVDF) membranes (EMD Millipore, Billerica, MA, USA) and subjected to western blotting using an antibody against ENO1.

## Additional Information

**How to cite this article:** Wang, R. *et al*. The Adhesion of *Lactobacillus salivarius* REN to a Human Intestinal Epithelial Cell Line Requires S-layer Proteins. *Sci. Rep.* **7**, 44029; doi: 10.1038/srep44029 (2017).

**Publisher's note:** Springer Nature remains neutral with regard to jurisdictional claims in published maps and institutional affiliations.

## Supplementary Material

Supplementary Information
