# Supplementary material for: The Adhesion of Lactobacillus salivarius REN to a Human Intestinal Epithelial Cell Line Requires S-layer Proteins
Source: Sci Rep. 2017 Mar 10;7:44029. doi: 10.1038/srep44029 (PMC5345100; doi:10.1038/srep44029)
Supplement: Supplementary Information [file srep44029-s1.pdf]

The Adhesion of *Lactobacillus salivarius* REN to a Human Intestinal Epithelial Cell Line Requires S-layer Proteins

Ran Wang [1#], Lun Jiang [1#], Ming Zhang[2], Liang Zhao[1], Yanling Hao[1], Huiyuan Guo[1], Yue Sang[3], Hao Zhang[1] & Fazheng Ren[1*]

[1]The Innovation Centre of Food Nutrition and Human Health (Beijing), College of Food Science and Nutritional Engineering, China Agricultural University, Beijing 100083, P. R. China

[2]School of Food and Chemical Engineering, Beijing Technology and Business University, Beijing 100048, P. R. China

[3]Beijing Higher Institution Engineering Research Center of Animal Product, Beijing 100083, P. R. China

# R Wang and L Jiang contributed equally to this work

[*]To whom correspondence should be addressed.

Fazheng Ren, China Agricultural University, Beijing 100083, P. R. China.

Tel.: 86-010-62736344; fax: 86-010-62736344.

Email: renfazheng@263.net

Supplemental Data

This article contains Supplemental Fig. S1 and Table S1, S2 and S3.

Supplemental Figure S1. The electrostatic potential map of CbpA and ENO1.

Supplemental Figure S2. The sequence analysis of SlpA, SlpB and RlpA.

Supplemental Figure S3. Enolase (ENO1), the putative CbpA receptor of HT-29 cells.

Supplemental Figure S4. Original gel drawing of PCR, SDS-PAGE and Western blotting.

Supplemental Table S1. Bacterial strains and plasmids used in this study.

Supplemental Table S2. Data collection and refinement statistics of CbpA.*

Supplemental Table S3. The list of primers used in the study.

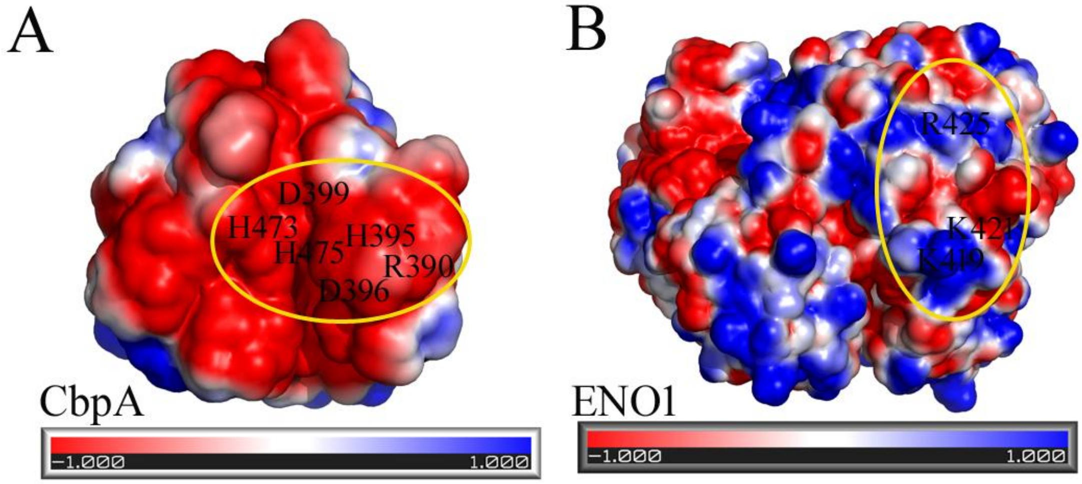

Supplemental Figure S1. The electrostatic potential map of **A.** CbpA and **B.** ENO1 (PDB ID: 3B97). The active site of CbpA and the C-terminal of ENO1 are shown in yellow ellipse. The key residues involved in the interaction between CbpA and ENO1 are labeled.

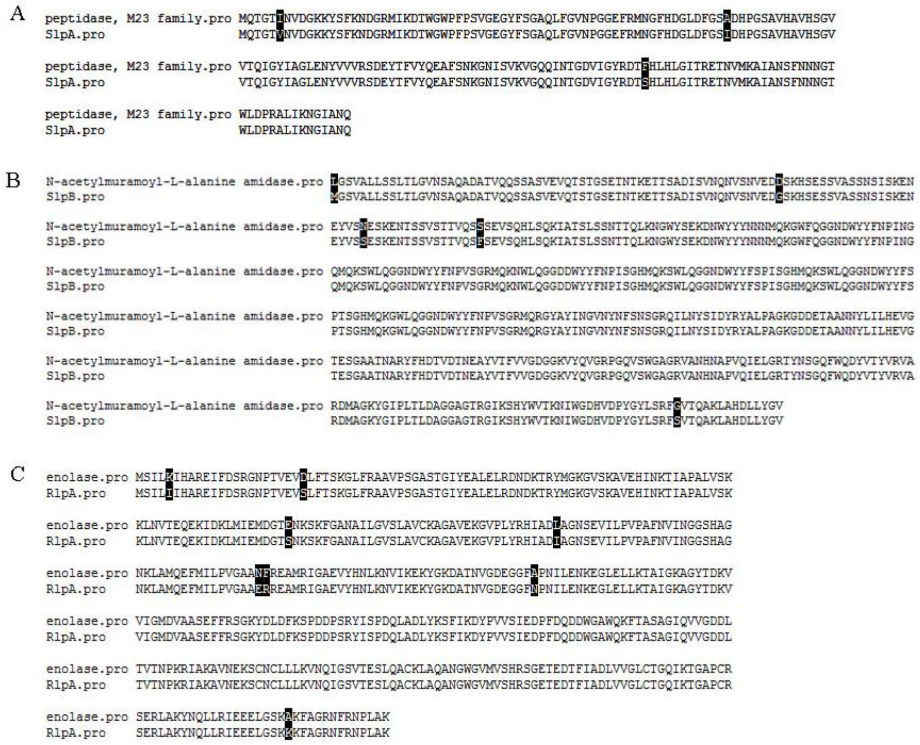

Supplemental Figure S2. The sequence analysis of SlpA, SlpB and RlpA. **A.** The multiple-amino-acid sequences alignment of SlpA with M23 peptidase of *L. salivarius*. **B.** Sequences alignment of SlpB with N-acetylmuramoyl-L-alanine amidase of *L. salivarius*. **C.** Sequences alignment of RlpB with enolase. Megalign program of LaserGene was used to perform the sequences alignment.

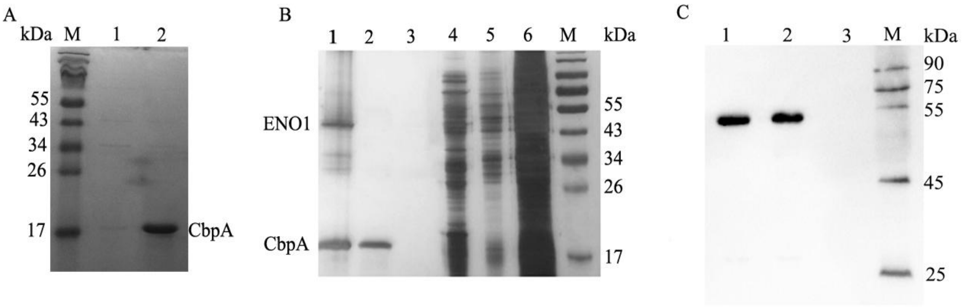

Supplemental Figure S3. Enolase (ENO1), the putative CbpA receptor of HT-29 cells. A. Recombinant CbpA was purified using Ni^{2+}-agarose affinity chromatography. Lane M: marker. Lane 1: flow-through liquid after purification with Ni^{2+}-agarose affinity chromatography. Lane 2: purified CbpA. **B.** The receptor of His-CbpA on HT-29 cells was identified using a His pull-down assay. Lane 1: bait protein (His-CbpA)-prey protein complex. Lane 2: bait protein (His-CbpA). Lane 3: control without bait protein. Lane 4: flow-through bait after binding to glutathione agarose. Lane 5: flow-through prey after incubation with immobilized bait. Lane 6: lysate of HT-29 cells. **C.** Western blotting analysis with anti-ENO1 polyclonal antibodies. Extracts from HT-29 cells were co-immunoprecipitated by anti-CbpA antibody. Co-immunoprecipitated proteins were analyzed by Western blotting with anti-ENO1 polyclonal antibodies. Lane 1: the receptor of His-CbpA. Lane 2: the receptor of CbpA. Lane 3: the control agarose resin.

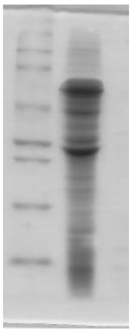

Figure. 2A

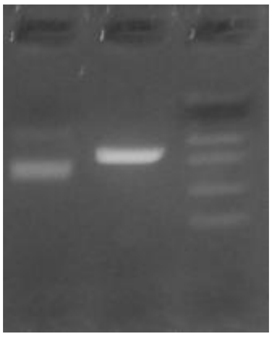

Figure. 2D

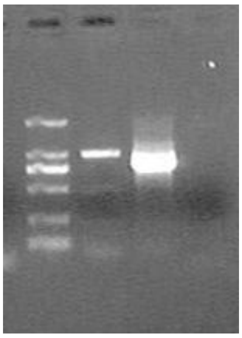

Figure. 2D

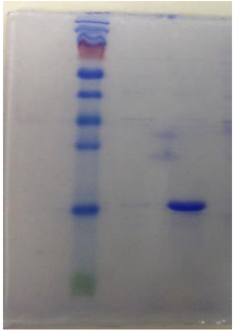

Figure. 6A

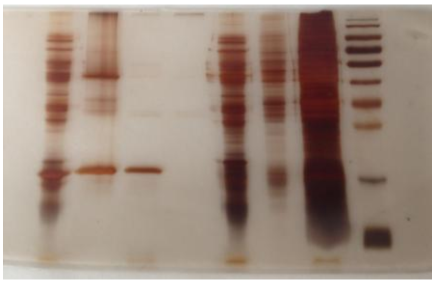

Figure. 6B

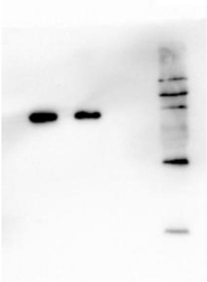

Figure. 6C

Supplemental Figure S4. Original gel drawing of PCR, SDS-PAGE and Western blotting.

Table S1. Bacterial strains and plasmids used in this study.

Bacterial strains or plasmids	Relevant properties	Reference of source
L. salivarius REN	Isolated from fecal samples of healthy centenarians	[1]
L. salivarius REN Δ *cbpA*	*L. salivarius* REN with deletion of the *cbpA* gene	This study
L. salivarius REN Δ *nam-amidase*	*L. salivarius* REN with deletion of the *nam-amidase* gene	This study
E. coli EC1000	Host for pORI19, chromosomal *repA*+ (pWV01)	[2]
pORI19	Em^r Ori^+ *repA*$^-$ *lacZ*’ derivative of pORI28	[2]
pTRK669	Temperature sensitive-helper plasmid, *repA*$^+$, Cm^r	[3]
pORS001	pORI19 containing flanks of *cbpA*	This study
pORS002	pORI19 containing flanks of *NAM-amidase*	This study
pET28b+	Expression vector	This study

Table S2. Data collection and refinement statistics of CbpA.*

	CbpA (PDB: 5GT1)
**Data collection**	
Wavelength	1.1000
Space group	*P*43212
Cell dimensions	
a,b,c (Å)	95.607, 95.607, 39.208
α,β,γ (˚)	90.0, 90.0, 90.0
Resolution (Å)†	50.0-1.85 (1.88-1.85)
*R*sym (%)	9.5 (45.9)
I/σ	37.0 (3.4)
Completeness (%)	93.0 (57.2)
Total No. of reflections	415874
Unique reflections	16222
Redundancy	15.9 (5.2)
**Refinement**	
Resolution (Å)	50.0-1.85 (1.85-1.90)
No. of reflections	14186 (598)
R_{work}/R_{free}(%)	17.2/19.7 (23.8/29.7)
No. of atoms	
Protein	1171
Ligand/ion	9
Water	141
B-factors(Å^2)	
Protein	19.41
Ligand/ion	31.20
Water	30.60
rms deviations	
Bond lengths (Å)	0.009
Bond angles (˚)	1.19
Ramachandran Plot (%)[1]	97.4/2.6/0.0/0.0

*Three crystal experiments for each structure.

$R_{sym}=\Sigma_h\Sigma_i|I_{h,i}-I_h|/\Sigma_h\Sigma_l I_{h,i},$ where I_h is the mean intensity of the i observations of symmetry related reflections of h.

[1]Residues in most favored, additional allowed, generously allowed and disallowed regions of the Ramachandran plot.

Table S3. The list of primers used in the study. a: F or R indicates forward primer or reverse primer, b: the underlines stand for restriction enzyme cutting site, c: 5’ or 3’ indicates location of primer upstream or downstream of target gene.

primers[a]	Sequence (5’-3’)[b]	Restriction enzyme cutting site	Target[c]
AIE51F	CCGGAATTCCAAACAGGTACTGTCAATGTAGATG	*Eco*R I	5’ *lspA*
AI52R	CCACACCACTATGAACTGCCTCATCAGAACGAACTACT	-	5’ *lspA*
AI33F	GCAGTTCATAGTGGTGTGGAGTAGTTCGTTCTGATGAG	-	3’ *lspA*
AIB34R	CGCGGATCCCTGATTAGCAATTCCAT	*Bam*H I	3’ *lspA*
BIE51F	CCGGAATTCCAAAAAGGCTGGTTTCAG	*Eco*R I	5’ *lspB*
BI52R	TAGTCAATGGAGTAGTTCATCACCCTTACCTGCTGGT	-	5’ *lspB*
BI33F	AACTACTCCATTGACTAACCAGCAGGTAAGGGTGATG	-	3’ *lspB*
BIB34R	CGCGGATCCCACACCATACAATAAATCAT	*Bam*H I	3’ *lspB*
ORI19DF	GGAAATTATCGTGATCAACAAGTTTA	-	pORI19
ANF	CCCATGGGCCAAACAGGTACT	*Nco* I	*lspA*

Reference

1. Zhang, M., Qiao, X. W., Zhao, L., Jiang, L. & Ren, F. Z. *Lactobacillus salivarius* REN counteracted unfavorable 4-nitroquinoline-1-oxide-induced changes in colonic microflora of rats. *J Microbiol* **49**, 877-883 (2011).

2. Law, J. *et al.* A system to generate chromosomal mutations in *Lactococcus lactis* which allows fast analysis of targeted genes. *J Bacteriol* **177**, 7011-7018 (1995).

3. Russell, W. M. & Klaenhammer, T. R. Efficient system for directed integration into the *Lactobacillus acidophilus* and *Lactobacillus gasseri* chromosomes via homologous recombination. *Applied and environmental microbiology* **67**, 4361-4364 (2001).
